# Silencing long non-coding RNA XIST suppresses drug resistance in acute myeloid leukemia through down-regulation of MYC by elevating microRNA-29a expression

**DOI:** 10.1186/s10020-020-00229-4

**Published:** 2020-11-24

**Authors:** Chong Wang, Lingling Li, Mengya Li, Weiqiong Wang, Yanfang Liu, Shujuan Wang

**Affiliations:** Department of Hematology, The First Affiliated Hospital of Zhengzhou University, No. 1, Jianshe East Road, Zhengzhou, 450052 Henan P. R. China

**Keywords:** Long non-coding XIST, microRNA-29a, Myelocytomatosis oncogene, Acute myeloid leukemia, Drug resistance

## Abstract

**Background:**

Long non-coding RNAs (lncRNAs) are biomarkers participating in multiple disease development including acute myeloid leukemia (AML). Here, we investigated molecular mechanism of X Inactive-Specific Transcript (XIST) in regulating cellular viability, apoptosis and drug resistance in AML.

**Methods:**

XIST, miR-29a and myelocytomatosis oncogene (MYC) expression in AML bone marrow cells collected from 62 patients was evaluated by RT-qPCR and Western blot analysis. Besides, the relationship among XIST, miR-29a and MYC was analyzed by dual luciferase reporter assay, RIP, and RNA pull down assays. AML KG-1 cells were treated with anti-tumor drug Adriamycin. The role of XIST/miR-29a/MYC in cellular viability, apoptosis and drug resistance in AML was accessed via gain- and loss-of-function approaches. At last, we evaluated role of XIST/miR-29a/MYC on tumorigenesis in vivo.

**Results:**

XIST and MYC were up-regulated, and miR-29a was down-regulated in AML bone marrow cells. Silencing XIST inhibited cellular activity and drug resistance but promoted cellular apoptosis of KG-1 cells by down-regulating MYC. XIST inhibited miR-29a expression to up-regulate MYC. Moreover, silencing XIST inhibited tumorigenesis of AML cells in vivo.

**Conclusions:**

Overall, down-regulation of XIST decreased MYC expression through releasing the inhibition on miR-29a, thereby reducing drug resistance, inhibiting viability and promoting apoptosis of AML cells.

## Background

Acute myeloid leukemia (AML) is a common aggressive bone marrow malignancy distinguished by deregulated proliferation and impaired differentiation of immature myeloid cells (Corces et al. 2017; Karjalainen and Repasky 2016). In recent years, deep understanding of the molecular and cytogenetic heterogeneity of AML has improved patient outcomes (Brinda et al. 2018). However, the prognosis of AML is still poor, and even younger patients receiving the strongest anti-leukemia treatment showed higher risk of recurrence for resistance to chemotherapy (Bruserud et al. 2017). In view of this, it is crucial to study the mechanisms underlying drug resistance to find new therapies to promote AML treatment.

Long non-coding RNAs (lncRNAs) can mediate resistance to anti-cancer drug by modulating DNA repair, drug efflux, and cellular apoptosis in cancer cells (Chen et al. 2017). Different lncRNAs are abnormally expressed in AML, which is related to the pathogenesis of AML (Lei et al. 2018). LncRNA X Inactive-Specific Transcript (XIST) is overexpressed in various cancers, suggesting that XIST may function as marker for cancer diagnosis (Sun et al. 2017; Zhu et al. 2018).

On the other hand, miRNAs involve in proliferation, apoptosis and drug resistance of cancer cells (Dehghanzadeh et al. 2015; Wu 2010). Some miRNAs are abnormally expressed in AML (Wallace and O'Connell 2017). For example, miR-29a is poorly expressed in AML, playing a regulatory role in bone marrow differentiation (Eyholzer et al. 2010; Wang et al. 2012). Moreover, myelocytomatosis oncogene (MYC) inhibits miR-29b expression in acute promyelocytic leukemia cells and participates in the mediation of miR-29b in bone marrow differentiation (Batliner et al. 2012). MYC proteins can modulate cell proliferation, survival, and metabolism by regulating gene expression (Hydbring et al. 2017). MYC is highly expressed in AML, in association with its drug resistance and poor prognosis (Li et al. 2014; Ohanian et al. 2019). This study attempts to provide theoretical support for AML by investigating XIST, miR-29a and MYC roles in AML cell functions.

## Methods

### Ethics statement

Study protocols were approved by Ethic Committee of The First Affiliated Hospital of Zhengzhou University and conducted in strict accordance with the *Declaration of Helsinki*. All participants signed informed consent documentation. Animal experiments accorded with the Guide for the Care and Use of Laboratory animals published by the US National Institutes of Health and approved by the Institutional Animal Care and Use Committee of The First Affiliated Hospital of Zhengzhou University (Zhengzhou, Henan Province, China).

### Bone marrow sample collection and cell culture

Bone marrow samples were obtained from 62 patients diagnosed with AML (38 males and 24 females, aged 10–52 years with a mean age of 31.98 ± 9.98 years) by the morphologic, immunologic, cytogenetic and molecular biologic classification in the Department of Hematology of The First Affiliated Hospital of Zhengzhou University (Zhengzhou, Henan Province, China) from September 2014 to September 2017. In addition, bone marrow samples were also collected from 20 healthy people and used as the control. According to French-American-British classification systems, there were 16 cases of M_2_, 24 of M_3_, 12 of M_4_, and 10 of M_5_. Complete blood counts revealed that the white blood cell count was 11.2–96.0 × 10^9^/L, platelet count was 31.2–86.1 × 10^9^/L, and hemoglobin level was 5.22–11.97 g/dL. Percentage of immature myeloid cells in bone marrow was 13.44–75.50%. Bone marrow mononuclear cells (BMNCs) were separated from bone marrow samples.

KG-1 cells (American Type Culture Collection, Manassas, VA, USA) were cultured in RPMI 1640 medium at 37 °C with 5% CO_2_. Cell growth was observed daily with an inverted microscope, and cells were passaged once every 2–3 days (Kobayashi et al. 2020).

### Cell treatment

KG-1 cells (3 × 10^5^ cells/mL) were seeded in a 6-well plate and transfected with si (small interfering RNA)-XIST, miR-29a mimic, or si-XIST + miR-29a inhibitor as well as their corresponding negative control (NC). miR-29a mimic and inhibitor were purchased from Genepharma (Shanghai, China) and others were purchased from Dharmacon, Lafayette, CO, USA).

### Reverse transcription quantitative polymerase chain reaction (RT-qPCR)

Extracted total RNA was reversely transcribed. RT-qPCR was carried out using SYBR® Premix Ex Taq™ II Kit (TaKaRa, Dalian, Liaoning, China) on an ABI 7500 instrument (Applied Biosystems, Foster City, CA, USA). The fold changes were calculated based on the 2^−ΔΔCt^ method. Gene expressions were relative to GAPDH while U6 served as the internal control of miR-29a. All primers were synthesized by Sangon Biotech Co., Ltd., (Shanghai, China) (Table 1).

**Table 1:** Primer sequences for RT-qPCR

Targeted genes	Forward sequence (5′–3′)	Reverse sequence (5–3′)
XIST	TCCTTTTCTGGCATCAGCGTT	GGCATCACCTCCTGGTTGAAT
miR-29a	TGCGCTAGCACCATCTGAAAT	CAGTGCAGGGTCCGAGGT
MYC	GGCTCCTGGCAAAAGGTCA	CTGCGTAGTTGTGCTGATGT
MRP1	TTCCGGAACTACTGCCTGCGCTA	GGGTCCTGGGGGATGATGGTG
P-GP	CCCATCATTGCAATAGCAGG	GTTCAAACTTCTGCTCCTGA
U6	CTCGCTTCGGCAGCACA	AACGCTTCACGAATTTGCGT
GAPDH	GGAGCGAGATCCCTCCAAAAT	GGCTGTTGTCATACTTCTCATGG

RT-qPCR, reverse transcription quantitative polymerase chain reaction; MRP1, multidrug resistance-associated protein 1, P-gp, P-glycoprotein; Bcl-2, B-cell leukemia/lymphoma 2, GAPDH, glyceraldehyde-3-phosphate dehydrogenase

### Western blot analysis

Proteins were isolated and then separated by sodium dodecyl sulfate polyacrylamide gel electrophoresis and transferred onto a nitrocellulose membrane, which was then incubated with rabbit anti-MYC (ab32072, 1: 10,000), MRP1 (ab180960, 1: 1000), P-GP (ab103477, 1: 500), Bcl-2 (ab59348, 1: 1000), Cleaved-caspase-3 (ab2302, 1: 1000), or GAPDH (ab9485, 1: 2500) at 4 °C overnight. Membrane was further incubated with diluted horseradish peroxidase-labeled secondary antibody, goat anti-rabbit immunoglobulins (IgG) (ab205718, 1: 2000–1: 50,000) for 2 h. The protein bands were visualized by enhanced chemiluminescence and photographed. Gray value ratio was analyzed using Quantity One software.

### Fluorescence in situ hybridization (FISH)

The FISH probe used in this experiment was synthesized by Sangon Biotech Co., Ltd. (Shanghai, China). Cells were inoculated in 24-well plates at a density of 6 × 10^4^ cells/well. Cells were fixed, blocked at 37ºC for 30 min by 20 µL pre-hybridization solution, and incubated with hybridization solution containing probes at 37ºC overnight. Cells were washed by lotion I for 3 times, by lotion II for 1 time and by lotion III for 1 time at 42ºC in dark. Finally, cells were stained with 4′,6-Diamidino-2-Phenylindole (DAPI) for 10 min and sealed.

### Dual luciferase reporter assay

The wild type (WT) sequences of XIST and MYC 3′untranslated region (3′UTR) along with the mutant (MUT) sequence in which miR-29a binding sites were mutated were artificially synthesized. Synthesized WT and MUT fragments were inserted into pmiR-RB-REPORT™ vector. WT and MUT reporter plasmid were co-transfected with miR-29a mimic or mimic NC (negative control) into 293 T cells respectively. The luciferase assay kit (RG005, Beyotime Institute of Biotechnology Co., Ltd., Shanghai, China) was used for determination of relative luciferase activity.

### RNA binding protein immunoprecipitation (RIP)

KG-1 cells were lysed and centrifuged. A part of cell extract was used as input, and the remaining was incubated with rabbit anti-argonaute 2 (AGO2; ab186733, 1: 50, Abcam Inc., Cambridge, UK) or rabbit anti-IgG (ab109489, 1: 100, Abcam Inc.) and magnetic beads at 4 °C overnight. The samples were placed on a magnetic base to collect immunoprecipitated complexes. RNA was extracted after treated with protease K for following PCR analysis (Zhang et al. 2018).

### RNA pull down assay

KG-1 cells were treated with 50 nM biotinylated Bio-probe NC, Bio-XIST-WT and Bio-XIST-MUT (Genecreate Bioengineering Co., Ltd., Wuhan, Hubei, China). After 48 h, cells were harvested and lysed. The cell lysate was incubated with M-280 streptavidin magnetic beads at 4 °C for 3 h. The bound RNA was purified by Trizol, and miR-29a enrichment was detected by RT-qPCR.

### Cell counting kit-8 (CCK-8)

For cell viability: KG-1 cells were dispersed into a single cell suspension, seeded in a 96-well plate at a density of 2.5 × 10^3^ cells/well. 24 h, 48 h and 72 h later, 10 μL of CCK-8 reagent (CK04, Dojindo Laboratories, Kumamoto, Japan) was added. The optical density (OD) at 450 nm was measured.

For drug resistance: KG-1 cells (2.5 × 10^3^ cells/well) were treated with Adriamycin at different concentration (5–100 μg/mL). After 48 h, 10 μL of CCK-8 reagent (CK04, Dojindo Laboratories, Kumamoto, Japan) was added to the cells and incubated for 2 h at 37 °C in a 5% CO_2_ incubator. OD value was then measured using an automatic microplate reader (Multiskan MK3, Thermo Fisher Scientific Inc.). The semi-inhibitory concentration (IC) 50 was calculated by the SPSS 22.0 statistical software (Li et al. 2020).

### Flow cytometry

KG-1 cells were collected and suspended in 1 × Binding Buffer, incubated with Annexin V-fluorescein isothiocyanate (FITC) for 15 min and Propidium Iodide (PI) for 5 min on ice bath. The cell cycle distribution was detected by flow cytometer (Cube6, Partec GmbH, Munster, Germany) at 480 nm. FITC was detected at 530 nm, and PI was detected at 575 nm.

For cell cycle, KG-1 cells were dispersed into single cell suspension, fixed in pre-cooled 70% ethanol overnight at 4 °C and centrifuged. Cells were incubated with RNase (1 mg/mL) at 37 °C for 30 min, then incubated with PI staining solution (50 μg/mL) for 40 min at 4 °C, followed by analysis with flow cytometry at wavelength > 575 nm.

### Xenograft tumor in nude mice

A total of 36 male 5-week-old BALB/c nude mice (18–20 g) from Shanghai Experimental Animal Center of Chinese Academy of Sciences (Shanghai, China), were fed with autoclaved standard laboratory feed and given free access to sterile drinking water. The cells were infected with lentiviruses carrying si-XIST, oe (overexpression)-MYC or si-XIST + oe-MYC or si-XIST + miR-29a inhibitor. Then the stable cells in the exponential growth phase were collected and made into single cell suspension at a density of 1 × 10^7^ cells/mL, 100 μL of which was inoculated subcutaneously into the dorsal root of the right hind limb of nude mice (1 × 10^6^ cells/mouse). Accordingly, the long diameter (a) and short diameter (b) of the tumor were measured and recorded every 3 days, the volume (v) was calculated: v = (a × b^2^)/2, and a growth curve was drawn. All mice were euthanized 30 days after inoculation, and tumors were excised and weighed.

### Statistical analysis

Measurement data were expressed as mean ± standard deviation. Differences between two groups were compared by unpaired *t* test. Comparisons among multiple groups following normal distribution were assessed by one-way ANOVA, followed by Tukey’s post-hoc test while data in skewed distribution among multiple groups were compared by Kruskal–Wallis test, followed by the Dunn's post-hoc tests. Tumor volume at different time points were analyzed by repeated measures ANOVA while proliferation ability at different time points was analyzed using two-way ANOVA. Enumeration data were presented as case number and analyzed using chi-square test. The correlation of XIST to miR-29a and MYC was analyzed using Pearson correlation analysis. *P* < 0.05 was statistically significant.

## Results

### XIST was highly expressed in AML bone marrow cells while its inhibition suppressed AML bone marrow cell proliferation and enhanced cell apoptosis and Adriamycin sensitivity

**Fig. 1:**
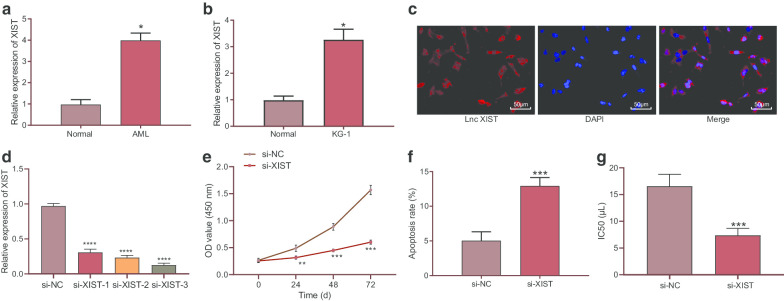
XIST was up-regulated in AML bone marrow cells while its silencing disrupted cell proliferation and enhanced cell apoptosis and Adriamycin sensitivity. **a** RT-qPCR was used to determine the expression of XIST in normal (n = 20) and AML bone marrow samples (n = 62). **b** RT-qPCR was used to determine the relative expression of XIST in KG-1 cells. **c** The subcellular localization of XIST in KG-1 cells identified by FISH assay. Red indicates XIST and blue represents nucleus. **d** Silencing efficiency of XIST in KG-1 cells assessed by RT-qPCR. **e** CCK-8 assay was used to detect the proliferation of KG-1 cells. F, The apoptosis of KG-1 cells detected by flow cytometer. **g** The sensitivity of KG-1 cells to Adriamycin. *p < 0.05 vs. normal bone marrow samples, normal bone marrow cell lines or si-NC-transfected cells. **p < 0.01 vs. si-NC-transfected cells. ***p < 0.001 vs. si-NC-transfected cells. The above data were measurement data, and expressed as mean ± standard deviation. The data between two groups were analyzed by unpaired *t*-test. The sensitivity of KG-1 cells to Adriamycin detected by CCK-8 assay at different time points was analyzed using two-way ANOVA. The experiment was repeated 3 times independently

The expression of XIST in AML bone marrow samples was first detected using RT-qPCR. The results showed a higher XIST expression in AML bone marrow samples than normal bone marrow samples (*p* < 0.05) (Fig. 1a). Besides, the clinical characteristics of patients with high or low XIST expression are presented in Table 2. It was found that the proportion of AML M3 was higher in patients with low XIST expression than in patients with high XIST expression (*p* < 0.05). High XIST expression in AML patients was correlated to more advanced risk stratification (*p* < 0.05). In addition, an upward trend was also observed in XIST expression in AML bone marrow KG-1 cell lines compared to normal bone marrow cell lines (*p* < 0.05) (Fig. 1b). FISH results showed that XIST was mainly localized in the cytoplasm (Fig. 1c). Next, the effect of XIST on the function of AML cells was further investigated. KG-1 cells were treated with si-XIST-1, si-XIST-2 and si-XIST-3, of which si-XIST-3 showed a superior silencing efficiency and therefore was used for subsequent experiments (Fig. 1d). CCK-8 and flow cytometry results revealed that the proliferation of KG-1 cells was reduced, while the apoptosis was increased upon transfection with XIST silencing (*p* < 0.05) (Fig. 1e, f). In addition, the sensitivity of KG-1 cells to Adriamycin was enhanced (*p* < 0.05) (Fig. 1g).

**Table 2:** Comparison of clinical and genetic features between XIST-high and XIST-low groups

	XIST		*P*
	High (n = 31)	Low (n = 31)	
Age	30.23 ± 9.45	33.74 ± 10.35	0.1683
White blood cell	31.6 ± 17.29	41.56 ± 26.51	0.0849
Platelet	69.75 ± 10.97	64.46 ± 13.12	0.0902
Hemoglobin	8.83 ± 1.57	8.37 ± 2.04	0.3238
FAB			0.0018
M_3_	6	18	
Non-M_3_	25	13	
Risk stratification of cytogenetics			0.0173
Good	5	15	
Intermediate	11	9	
Poor	15	7	

XIST, X Inactive-Specific Transcript; FAB, French-American-British

### XIST could competitively bind to miR-29a to regulate MYC expression

**Fig. 2:**
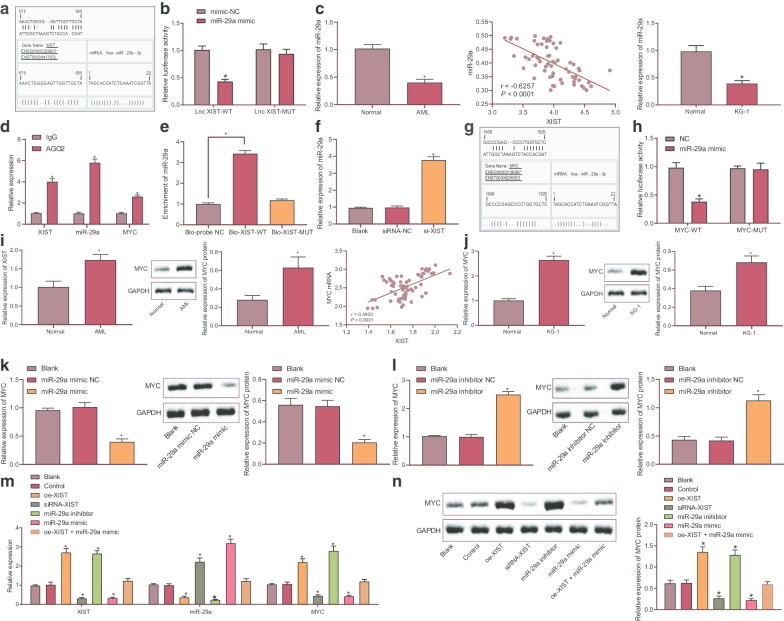
XIST could competitively bind to miR-29a and up-regulate MYC expression. **a** The binding sites between miR-29a and XIST predicted by the online analysis. **b** The binding of miR-29a to XIST confirmed by dual luciferase reporter assay. **c** The relative expression of miR-29a in AML bone marrow samples determined by RT-qPCR (left); correlation between miR-29a and XIST analyzed by Pearson correlation analysis (middle); the expression of miR-29a in normal bone marrow mononuclear cells and KG-1 cells (right). **d** RIP was used to analyze the binding of AGO with XIST, MYC, miR-29a. **e** RNA pull down was used to analyze the binding of XIST with miR-29a. **f** RT-qPCR was used to determine the expression miR-29a in cells upon varied treatments. **g** The predicted binding sites between miR-29a and MYC. **h** Putative miR-29a binding site in the 3′UTR region of MYC confirmed by dual luciferase reporter assay. **i** RT-qPCR and Western blot analysis were used to determine the relative expression of MYC in AML bone marrow samples; correlation of XIST to MYC determined by Pearson correlation analysis (middle). **j** RT-qPCR and Western blot analysis were used to determine the relative expression of MYC in KG-1 cells. **k** RT-qPCR was used to determine the expression of MYC in cells overexpressing miR-29a. **l** RT-qPCR and Western blot analysis were used to determine the relative expression of MYC in cells with miR-29a knockdown. **m** RT-qPCR was used to determine the expression of XIST, miR-29a and MYC in cells upon varied treatments. **n** Western blot analysis was used to determine the protein expression of MYC in cells upon varied treatments. *p < 0.05 vs. KG-1 cells without any treatment. The data were measurement data, and expressed as mean ± standard deviation. Data comparison among multiple groups was conducted by one-way ANOVA. The experiment was repeated 3 times independently

Bioinformatics analysis was subsequently carried out using relevant databases in order to explore the potential downstream regulatory targets of XIST. The online prediction database Jefferson (https://cm.jefferson.edu/rna22/Interactive/) predicted binding sites between miR-29a and XIST (Fig. 2a). The predicted results were further validated using dual luciferase reporter assay, which showed decreased luciferase activity of XIST-WT upon miR-29a mimic transfection (*p* < 0.05) while no changes were observed in the luciferase activity of XIST-MUT (*p* > 0.05) (Fig. 2b). In addition, RT-qPCR results revealed a lower miR-29a expression in AML bone marrow samples than normal bone marrow samples, which was negatively associated with the expression of XIST (*p* < 0.05) (Fig. 2c). It is widely known that miRNA might form RNA-induced silencing complex (RISC), during which miRNA is coated with AGO protein, mainly AGO2. RIP assay showed that AGO2 antibody could precipitate XIST, miR-29a and MYC (*p* < 0.05) (Fig. 2d), indicating that XIST could form a complex with AGO2. RNA pull down assay showed that miR-29a enrichment was increased in Bio-XIST-WT (*p* < 0.05) but it exhibited no changes in Bio-XIST-MUT (*p* > 0.05) (Fig. 2e), suggesting the competitive binding of XIST to miR-29a and the ability to regulate miR-29a. Furthermore, RT-qPCR results displayed up-regulated miR-29a expression upon silencing XIST (*p* < 0.05) (Fig. 2f). These results supported the notion that XIST could specifically target miR-29a and inhibit its expression.

As depicted in Fig. 2g, there were binding sites between miR-29a and MYC 3′UTR. Dual luciferase reporter assay further verified their binding, which was reflected by reduced luciferase activity of MYC-WT upon miR-29a mimic transfection (*p* < 0.05) while no difference was observed in the luciferase activity of MYC-MUT (*p* > 0.05) (Fig. 2h). Both AML bone marrow samples and KG-1 cells exhibited up-regulated expression of MYC, which was positively associated with the expression of XIST (*p* < 0.05) (Fig. 2i, j). Additionally, KG-1 cells overexpressing miR-29a presented with reduced MYC mRNA and protein expression, which was negated by miR-29a inhibition (*p* < 0.05) (Fig. 2k, l).

Subsequent results of RT-qPCR showed that overexpression of XIST decreased the expression of miR-29a while elevating the expression of MYC, and this trend was reversed by silencing of XIST. Inhibition of miR-29a resulted in an increase in MYC expression, whereas overexpression of miR-29a decreased MYC expression. Compared with overexpression of XIST alone, dual transfection with oe-XIST and miR-29a mimic reduced MYC; when in comparison with overexpression of miR-29a alone, co-treatment of oe-XIST and miR-29a mimic led to increased MYC; however, there was no changes in MYC expression following the co-treatment of oe-XIST and miR-29a mimic when in comparison with the control group (all *p* < 0.05) (Fig. 2m). In addition, Western blot analysis yielded similar results at the protein expression MYC (all *p* < 0.05) (Fig. 2n). The aforementioned data indicated that XIST could promote the expression of MYC through competitive binding of miR-29a.

### Silencing XIST inhibited the viability and reduced drug resistance of AML bone marrow cells via down-regulation of MYC

**Fig. 3:**
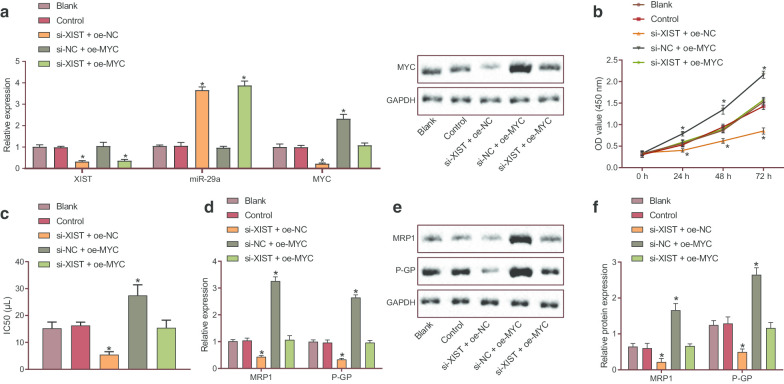
Down-regulated XIST suppressed the viability of KG-1 cells and decreased their resistance to Adriamycin via MYC down-regulation. **a** miR-29a expression and mRNA expression of XIST and MYC as well as MYC protein expression in KG-1 cells detected by RT-qPCR and Western blot analysis. **b** CCK-8 assay was used to detect the viability of KG-1 cells. **c** CCK-8 assay was used to detect the Adriamycin resistance of KG-1 cells. **d** RT-qPCR was used to examine the relative expression of MRP1 and P-GP in KG-1 cells. **e**, **f** Western blot analysis was performed to test the expression of drug-resistant proteins (MRP1 and P-GP) in KG-1 cells. *p < 0.05 vs. KG-1 cells without any treatment. The data were measurement data, and expressed as mean ± standard deviation. Data comparison among multiple groups was conducted by one-way ANOVA. Cell proliferation ability detected by CCK-8 assay at different time points was measured using two-way ANOVA. The experiment was repeated 3 times independently

In order to investigate the effect of XIST/miR-29a/MYC signaling axis on the development of AML and chemosensitivity, a series of molecular biological experiments were carried out on KG-1 cells. Silencing of XIST resulted in decreased expression of XIST and MYC yet increased miR-29a expression. In addition, MYC expression was up-regulated in the presence of MYC overexpression (all *p* < 0.05) (Fig. 3a). CCK-8 assay revealed decreased cell viability following transfection with silencing XIST at 24 h, 48 h and 72 h (*p* < 0.05), while it was augmented upon transfection with MYC overexpression or in combination with XIST silencing (*p* < 0.05) (Fig. 3b). Next, Adriamycin at different concentration (5–100 μg/mL) was used to treat KG-1 cells. The CCK-8 assay results showed that IC50 of anti-tumor drug doxorubicin on KG-1 cells was decreased (Fig. 3c), and drug-resistant proteins MRP1 and P-GP showed down-regulated expression (Fig. 3d–f) after silencing XIST, which was rescued by overexpression of MYC (all *p* < 0.05). These results suggested that silencing XIST inhibited the viability and increased the sensitivity of AML bone marrow cells to doxorubicin by down-regulating MYC.

### Silencing XIST promoted apoptosis of AML bone marrow cells via down-regulation of MYC

**Fig. 4:**
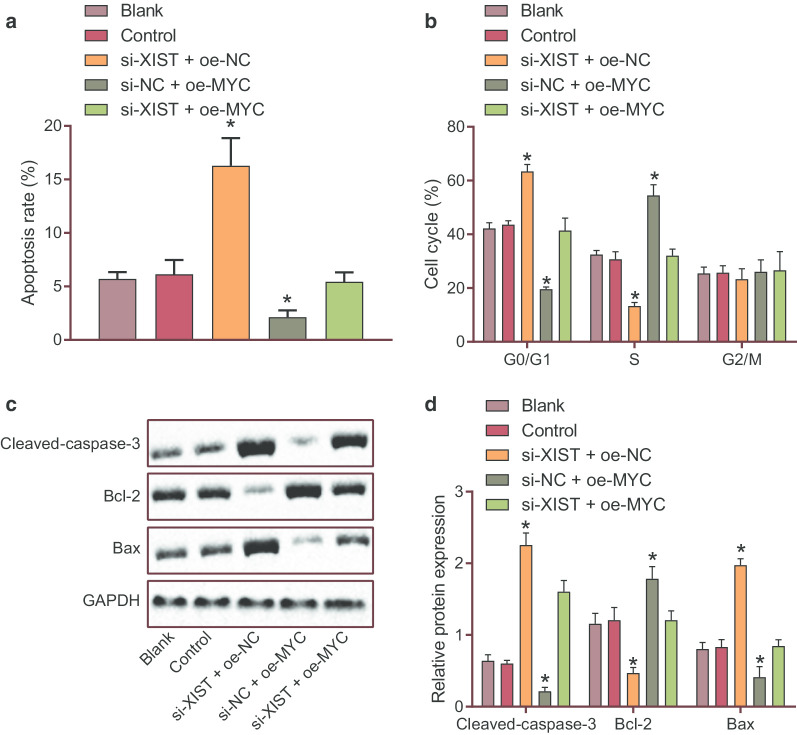
Down-regulated XIST enhanced apoptosis and slowed down cell cycle progression in KG-1 cells via MYC down-regulation. **a** The apoptosis of KG-1 cells detected by flow cytometry. **b** The cell cycle distribution of KG-1 cells examined by flow cytometry. **c**, **d** The expression of apoptosis-related genes (Bcl-2, Bax and Cleaved-caspase-3) in KG-1 cells determined by Western blot analysis. *p < 0.05 vs. KG-1 cells without any treatment. The data were measurement data, and expressed as mean ± standard deviation. Data comparison among multiple groups was conducted by one-way ANOVA. The experiment was repeated 3 times independently

We then proceeded to dissect out the effect of XIST/miR-29a/MYC signaling axis on the apoptosis of AML bone marrow cells. Flow cytometric analysis showed that the apoptosis rate of KG-1 cells was increased following silencing of XIST while it was diminished upon MYC overexpression. In addition to that, the apoptosis rate displayed a downward trend in the presence of both XIST silencing and MYC overexpression (*p* < 0.05) (Fig. 4a). The number of cells arrested in the G0/G1 phase was increased, while the number of cells arrested in the S and G1/G2 phases was decreased by inhibition of XIST (*p* < 0.05), which was abolished by MYC overexpression (*p* < 0.05). G0/G1 phase-arrested cells were much lower and the S and G1/G2 phases-arrested cells were more in response to both XIST silencing and MYC overexpression than XIST silencing alone (*p* < 0.05) (Fig. 4b). Furthermore, silencing XIST down-regulated the protein expression of anti-apoptotic gene Bcl-2 but up-regulated the protein expression of pro-apoptotic gene Cleaved-caspase-3 and Bax (*p* < 0.05) (Fig. 4c, d). Therefore, silencing XIST promoted apoptosis retarded cell cycle progression in AML bone marrow cells by decreasing MYC expression.

### Silencing XIST reduced tumorigenic ability of KG-1 cells in vivo by up-regulating miR-29a and down-regulating MYC

**Fig. 5:**
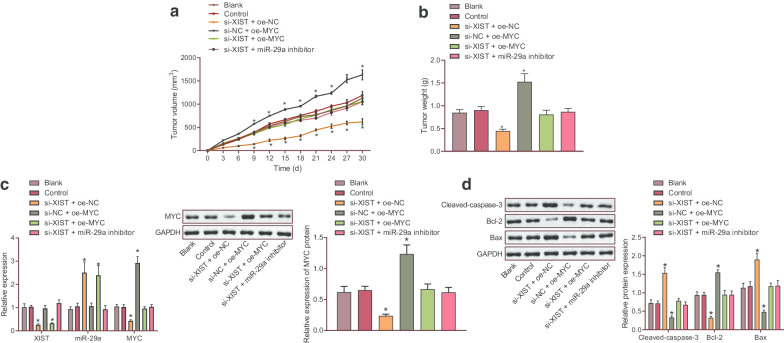
Down-regulated XIST elevated miR-29a and reduced MYC to inhibit tumorigenic ability of KG-1 cells in nude mice. **a** The volume of tumor in nude mice. **b** The weight of tumor in nude mice. **c**, **d** The expression of apoptosis-related genes (Bcl-2, Bax and Cleaved-caspase-3) in KG-1 cells determined by Western blot analysis. * p < 0.05 vs. nude mice bearing KG-1 cells treated with PBS alone or nude mice bearing KG-1 cells treated with si-NC + oe-NC. The data were all measurement data, and expressed as mean ± standard deviation. Tumor volume was analyzed by repeated measures ANOVA, and tumor weight was analyzed by one-way ANOVA, n = 6. The experiment was repeated 3 times independently

Xenograft tumors were developed in nude mice to detect the effect of XIST/miR-29a/MYC signaling axis on the AML progression in vivo. As illustrated in Fig. 5a, b, treatment with silencing XIST decreased tumor volume and weight, while overexpression of MYC reversed the trends (*p* < 0.05). Western blot analysis results demonstrated an increase in the protein expression of Cleaved-caspase-3 and Bax yet a reduction in Bcl-2 protein expression in the absence of XIST (*p* < 0.05), but this tendency was undermined by overexpression of MYC (*p* < 0.05). Additionally, compared with the treatment with silencing XIST, co-treatment of si-XIST and oe-MYC and co-treatment of si-XIST and miR-29a inhibitor resulted in increased tumor volume and weight, up-regulated expression of MYC and Bcl-2 but down-regulated expression of BAX and Cleaved-caspase 3 (Fig. 5c, d). Overall, these findings supported that inhibiting XIST could up-regulate miR-29a, down-regulate MYC, and inhibit the tumorigenic ability of KG-1 cells in nude mice.

## Discussion

In spite of extensive improvements in comprehending the risk stratification and biology, the resistance to chemotherapy is a considerable challenge for improving AML treatment (Cassier et al. 2017; Shaffer et al. 2012). Therefore, the discovery of novel therapeutic targets is critical for reducing the resistance of AML cells to drugs. The data obtained from the present study showed that silencing XIST could reduce drug resistance, inhibit proliferation, and promote apoptosis of AML cells through down-regulation of MYC induced by miR-29a.

XIST has been reported to function as an oncogene or a tumor suppressor in different human malignancies, which is implicated in many aspects of carcinogenesis including tumor apoptosis, cell cycle, initiation, invasion, metastasis, stemness, autophagy and drug resistance (Yang et al. 2018). XIST was found to be up-regulated in AML bone marrow cells. In addition, silencing of XIST could repress AML bone marrow cell proliferation while enhancing cell apoptosis and Adriamycin sensitivity. XIST is highly expressed in breast cancer and is closely associated with a poor prognosis and the resistance to chemotherapy (Schouten et al. 2016). In addition, the expression of XIST is significantly abundant in both in vivo and in vitro Alzheimer's disease models (Yue et al. 2020). XIST expression is also found to be up-regulated in human umbilical vein endothelial cells treated by oxidized low-density lipoprotein during atherosclerosis (Hu et al. 2019). Knockdown of XIST has been shown to inhibit glioma cell proliferation and induce cell apoptosis as well as enhancing cell sensitivity to X-ray radiation (Wang et al. 2020), which is partially accordance to our results. Down-regulation of XIST has been reported to reduce chemoresistance in non-small cell lung cancer cells by inhibiting autophagy (Sun et al. 2017). Knockdown of XIST can inhibit proliferation of osteosarcoma cells (Li et al. 2017). However, little is known about the role of XIST in AML, which merits further investigation.

XIST could specifically target miR-29a and inhibit its expression. Previous data suggested that XIST can inhibit expression of miR-29a by direct targeting in denatured dermis and human skin fibroblasts after thermal injury (Guo et al. 2018). In agreement with our findings, miR-29a was found to be down-regulated in peripheral blood mononuclear cells and modulated process of AML (Wang et al. 2012). Overexpression of miR-29a inhibits proliferation and promotes apoptosis of AML cells (Gong et al. 2014). Moreover, miR-29a regulates self-renewal and drug resistance of AML cells by reducing expression of Ski oncogene (Teichler et al. 2011). Furthermore, our study also provided evidence suggesting MYC to be a target of miR-29a. MYC is a transcription factor playing important roles in cellular processes, including cell cycle, proliferation, apoptosis, and differentiation (Mainwaring et al. 2010). MYC is elevated in AML cells and depletion of MYC can inhibit cell proliferation (Guo et al. 2014). Moreover, MYC has been highlighted to be a target gene of miR-29b, and inhibition of miR-29b impairs neutrophil differentiation of acute promyelocytic leukemia cells through regulating MYC expression (Batliner et al. 2012).

LncRNAs have been reported to exert their functions through acting as a ceRNA to sponge miRNAs, thereby regulating miRNA targeted gene expression (Militello et al. 2017). H19 interacting with miR-29b regulates progranulin expression, which mediates invasion, migration and apoptosis of colorectal cancer cells (Ding et al. 2018). H19 regulates AML cell proliferation through competitively binding to hsa-miR-19a/b and mediating inhibitor of DNA binding 2 (Zhao et al. 2017). Consistently, overexpression of XIST in denatured dermis augments expression LIN28A which is a target gene of miR-29a, by binding to miR-29a, thus promoting human skin fibroblast proliferation, migration and extracellular matrix (ECM) synthesis (Guo et al. 2018). Hence, based on the aforementioned information, we are convinced that silencing XIST may have the potential to inhibit the viability and reduce drug resistance of AML bone marrow cells via miR-29a-mediated MYC down-regulation.

## Conclusion

**Fig. 6:**
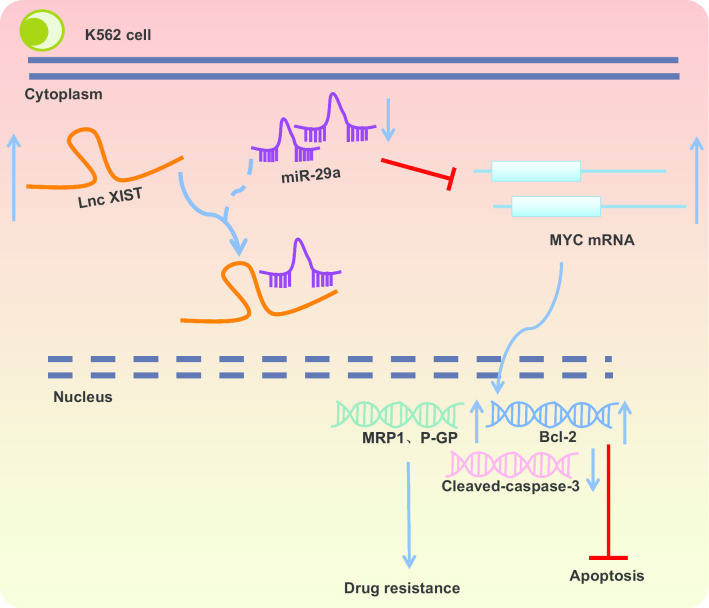
The molecular mechanisms illustrating the effect of the XIST/miR-29a/MYC axis on viability, apoptosis and drug resistance of AML cells. XIST can competitively bind to miR-29a to down-regulate miR-29a expression and up-regulate MYC expression, thereby increasing drug resistance and suppressing the apoptosis of AML cells

In conclusion, down-regulation of XIST promoted the down-regulation of MYC in AML through releasing inhibition on miR-29a, thereby reducing drug resistance, inhibiting proliferation and promoting apoptosis of AML cells (Fig. 6). At present, regulatory mechanism of the XIST/miR-29a/MYC axis remains scantly identified in AML, and we will further study underlying rules governing lncRNA-miRNA interaction.

## Data Availability

The datasets used and/or analyzed during the current study are available from the corresponding author on reasonable request.

## Abbreviations

lncRNAs: Long non-coding RNAs
AML: Acute myeloid leukemia
XIST: X Inactive-Specific Transcript
MYC: Myelocytomatosis oncogene
NC: Negative control
RT-qPCR: Reverse transcription quantitative polymerase chain reaction
FISH: Fluorescence in situ hybridization
DAPI: 4′,6-diamidino-2-phenylindole
WT: Wild type
3′UTR: 3′untranslated region
MUT: Mutant
RIP: RNA binding protein immunoprecipitation
CCK-8: Cell counting kit-8
OD: Optical density
IC: Inhibitory concentration
FITC: Fluorescein isothiocyanate
PI: Propidium iodide
